# Case report: Online eye movement desensitization and reprocessing approach in children: a case series

**DOI:** 10.3389/fpsyt.2024.1391980

**Published:** 2024-10-08

**Authors:** Canan Citil Akyol, Mustafa Kutlu, Ümran Korkmazlar

**Affiliations:** ^1^ Psychology Department, Cumhuriyet University, Sivas, Türkiye; ^2^ Psychological Counseling and Guidance Department, İnönü University, Malatya, Türkiye; ^3^ Independent researcher, İstanbul, Türkiye

**Keywords:** EMDR, trauma, psychological counseling, online, case study

## Abstract

**Objective:**

The aim of this study was to examine the use of Eye Movement Desensitization and Reprocessing (EMDR) in online counseling for children with single-incident trauma.

**Method:**

A qualitative case study method was employed. The research was conducted with two volunteering children and their parents. The sessions were transcribed by the authors, and code names (Ipek and Eylül) were assigned to protect the participants’ identities. İpek’s traumatic memory was explored through drawing pictures with tactile bilateral stimuli and Eylül’s traumatic experience was addressed using tactile bilateral stimuli during the EMDR therapeutic story technique. The Child Revised Impact of Events Scale (CRIES-8) was used for pre-test, post-test, and follow-up testing to support the session descriptions.

**Results:**

The results indicated that post-traumatic symptoms were reduced and remained at a low level for an extended period in both clients.

**Conclusions:**

It is suggested that future studies should explore various bilateral stimulation methods in online EMDR, conduct larger-scale studies with children who have experienced different types of traumatic events, and investigate the impact of various EMDR protocols on children.

## 1 Introduction

Childhood is a vulnerable and impressionable stage in life, making it susceptible to external influences. Research has shown that traumas such as parental divorce, loss of a loved one, natural disasters and child abuse can have a lasting impact on individuals (1–3). While there have been several studies on the effects of childhood problems on adolescents and adults, there is limited research specifically focused on problems experienced by children as a result of childhood experiences (4–6).

The 7 recommended EMDR therapy as an effective approach for psychological interventions related to traumatic experiences. Eye Movement Desensitization and Reprocessing is an 8-stage protocol used to alleviate the effects of traumatic experiences. The approach is based on the idea that traumatic events disrupt the normal processing of memories, leading to anxiety and negative self-perception. EMDR helps to change negative self-ideation and reduce the impact of disturbing traumatic images (8).

EMDR was first used for treating trauma in children by 9, called the ‘Developmental Protocol.’ It adapts the 8 stages of EMDR to the age and developmental level of the child (10, 11).

### 1.1 EMDR phases in children

The first phase, which includes gathering detailed information about the child’s developmental history, parental background, and any trauma they may have experienced, is called history taking and therapy planning (9). In the next stage, referred to as preparation, the child’s stability is assessed, and techniques such as safe place visualization, muscle relaxation, and breathing exercises are implemented to help those who are unable to process traumatic memories (12, 13). Phase three focuses on assessing trauma-related symptoms that involve vivid visual imagery associated with traumatic memories, positive and negative believe about self, relevant emotions, and bodily sensations. The worst image of the target memory is identified and the subjective discomfort level (SUD) is rated on a scale of 0-10. In EMDR, it is commonly used prior to the start of bilateral stimulation (BLS), and later to quantify the client’s report of reduced or eliminated disturbance and other treatments to measure baseline emotional or physical pain, as well as to assess progress being made (14). The validity of positive cognition (VOC) is also evaluated on a 7-point Likert type scale. The positive cognition is rated while thinking about the traumatic experience, with 1 = feeling totally false and 7 = feeling totally true. Additionally, somatosensory and emotional associations with the worst image are noted (12). The fourth phase, desensitization, can take a few minutes in one session or multiple sessions spanning several months. Desensitization involves a unique procedure where the therapist exposes the patient to bilateral visual (eye movement), auditory, or sensory stimulation (e.g., tactile stimulation) that includes BLS. Techniques such as drawings or stories may be used during this phase, tailored to the child’s developmental level (15). The installation phase is the fifth stage in EMDR therapy. Installation stage is initiated when the SUD level of the child is 0 during the desensitization phase and is used to ensure that the child has positive beliefs after the desensitization phase. The body scan stage, which is the sixth phase, is initiated when the child’s level of distress is low and involves the child scanning their body for any discomfort while thinking about the traumatic event in a positive way. If discomfort is present, the process continues with BLS, otherwise the debriefing and closure stage is initiated (12). This seventh phase aims to leave the child in a positive mental state and with self-confidence (8). The final phase is reevaluation, where the memories and symptoms are evaluated and a healthy future is planned. New symptoms should be assessed in interviews with parents using a holistic approach (12).

### 1.2 Online EMDR

EMDR is a unique therapy method that typically involves following rhythmic eye movements or tactile stimuli, making it difficult to conduct online. However, the COVID-19 pandemic has made online counseling an essential option, due to quarantines and curfews. A systematic review on the use of online EMDR for treating post-traumatic stress disorder during the pandemic showed that it can be effective for both adults and children. However, considering the limitation that the study findings are based on a single research, the results should be interpreted with caution (16). The current study aims to provide insight into the therapy’s effectiveness by describing actual cases of online EMDR sessions. Previous research has shown that online psychological counseling can be effective for treating depression, anxiety disorders, behavioral problems and phobia in children (17, 18). However, research on online EMDR therapy for children is relatively new. One of these studies, McGowan et al. (19), reported that EMDR therapy administered online to 93 clients during the COVID-19 pandemic was clinically effective in both adult and child/adolescent populations. Bursnall et al. (20) collected data on the online use of EMDR through surveys and interviews from a group of EMDR therapists (n=562) and their clients (n=148). Although the study results noted that internet connection interruptions could hinder the therapy process, 88% of the clients reported being extremely satisfied with receiving EMDR therapy online. Additionally, therapists expressed greater willingness to implement online EMDR therapy by the end of the year. Studies on the online use of EMDR indicate a need for more research to support whether EMDR is as effective as face-to-face therapy (21, 22). The current study aims to contribute to the literature by describing the effectiveness of online EMDR therapy for children, which may not have been considered a suitable candidate for online application. The purpose of this study is to examine the impact of online “Eye Movement Desensitization and Reprocessing (EMDR)” on children.

## 2 Methods

### 2.1 The research model

The current article uses the qualitative case study approach to examine the relationship established and interventions employed during counseling, as well as the client’s perceptions of these interventions. This approach emphasizes the uniqueness of individuals and is useful for analyzing the internal experiences of individuals without the limitations of measurable variables. The direct involvement of the individual in the research is a key strength of this method, and it is also well-suited for studying rare occurrences in psychological health. Additionally, this approach can be used to illustrate counseling practices, demonstrate the adopted psychological counseling approach in depth, and guide future studies. The differences between interventions can also be described using this method (23, 24). After obtaining ethical approval from the İnönü University Research and Publication Ethics Committee (Approval No: 2020/18-3), EMDR sessions started.

### 2.2 The clients

The study involved two children. One 6-year-old and another 8-year-old, who were both identified as at risk of PTSD by psychological counselors at their schools using the diagnostic criteria from the DSM-V. They were later confirmed to have PTSD by a psychiatrist and then were included in the study.

#### 2.2.1 First client

An 8-year-old girl named Ipek. Three years prior to the study, Ipek was traveling with her family when their car collided with a boar, resulting in an accident. Ipek was asleep at the time of the crash and woke up to the sight of her sister bleeding and the car being severely damaged. Since the accident, Ipek has been unable to travel in cars without experiencing severe distress and crying halfway through the trip. She also cannot stay home alone. Her parents sought psychological counseling to address her symptoms. Ipek’s score on the Child Revised Impact of Events Scale (CRIES-8) was 22 (High PTSD symptoms).

#### 2.2.2 Second client

A 6-year-old girl named Eylül. When she was 4 years old, Eylül learned that her grandmother had cancer and witnessed her grandmother’s chemotherapy treatment for 9 months, including the adverse effects of the treatment. Eylül was not told about her grandmother’s passing and only learned of it at her grandmother’s wake. Since then, Eylül has stopped talking about her grandmother, cries when someone mentions her, and avoids the topic altogether. She also has difficulty sleeping alone and is anxious about her parents growing old. Her mother sought psychological counseling to address Eylül’s symptoms such as introversion, silence, and insomnia due to the loss of her grandmother. Eylül’s score on the Child Revised Impact of Events Scale (CRIES-8) was 21 (High PTSD symptoms).

### 2.3 Data collection instruments

#### 2.3.1 Child Revised Impact of Events Scale

The Child Revised Impact of Events Scale (CRIES-8) was used in this study to assess the persistence of traumatic experiences and their effects after counseling sessions. CRIES-8 is a self-rating scale that uses a 4-point Likert-type system (Never=0, Rarely=1, Sometimes=3, Often=5) to screen for symptoms of post-traumatic stress disorder in children. It has been reported that a cut-off score of 17 on the CRIES-8 provides maximum sensitivity and specificity in detecting PTSD (25). Çeri et al. (26) stated that the scale yielded valid and reliable results in the Turkish culture. It has two sub-dimensions: intrusion (sample item: “Images about that event suddenly appear before my eyes”) and avoidance (sample item: “I try to stay away from places and situations that remind me of those events”) and the total scale score ranges from 0 to 40 (27).

#### 2.3.2 Diaries

In this study, diaries were used as a method of collecting qualitative data. This method is flexible and can be used to address various research problems. Keeping a diary is considered important in the analysis of clients’ emotions and thoughts in psychology (28). The parents of the children who received psychological counseling were asked to keep a diary, in which they recorded the emotions and positive/negative experiences of their children, as well as their own self-awareness as parents, after the sessions until the next interview. The researcher also kept a diary, the Psychological Counseling Diary (PCD), in which she recorded her own experiences, observations of problems and solutions encountered during the preparation for sessions or during online sessions and their relationship with the clients.

### 2.4 Data analysis

The current study focuses on describing the use of online EMDR therapy in two separate children diagnosed with PTSD. Diagnoses were made through an online interview by a psychiatrist. Also, sessions with the children were conducted via the Zoom platform, recorded, and transcribed by the researcher. During the writing of the findings, the article format categorized the data according to the eight phases of EMDR, and the transcripts were used to describe these phases. Since the phases were addressed in the introduction section, they were not repeated here. Parent diaries and psychological counselor diaries were also collected in Word documents created specifically for the participants. While parent diaries were used to gain in-depth insights into the participants’ perspectives, the psychological counselor diary provided observations, particularly regarding the implementation of EMDR over the internet. Additionally, CRIES-8 was used as a quantitative data tool in the study. Scores obtained from this scale were compared for each client based on pre-test, post-test, and follow-up test results. The aim was to support client statements and parent diary data rather than relying solely on numeric data.

### 2.5 Validity and reliability

#### 2.5.1 Validity

In this study, participating clients were observed and direct quotes from the clients were included to enhance the credibility of the findings. The transferability of the results, or their potential applicability in similar settings, was improved through detailed description and the use of purposive sampling (29). Additionally, session transcripts were shared with the participating parents to ensure accurate representation of the clients’ views and consent was obtained from the parents confirming that their statements matched the transcripts.

#### 2.5.2 Reliability

In the study, one-to-one counseling sessions (including silences and minimal prompts) were transcribed to provide a rich and detailed context. However, external reliability was low due to the uniqueness of each client and the fact that even if their problems are similar, the sessions are not identical. To improve external reliability, data collection limits were established and inclusion criteria were defined to control for client characteristics. To minimize bias, expert opinion from a supervisor was sought out in the study.

## 3 Findings

In this section, the EMDR sessions conducted with Ipek are described in detail. To avoid technical repetition, only detailed descriptions of the preparation and desensitization stages of the EMDR sessions conducted with Eylül are provided, while the remaining sessions are briefly mentioned.

### 3.1 Ipek’s sessions

The contents of the sessions are summarized in the relevant tables before describing İpek’s EMDR therapy process.

#### 3.1.1 History taking and therapy planning

Before moving to the preparation phase of EMDR with İpek, both parents were invited for an interview to gather the history taking. However, due to the father’s busy work schedule, he could not participate in the process. Instead, the mother attended a 60-minute online session. The session was conducted using the Zoom program. **Table 1** provides a summary of the process, followed by a description of the process.

**Table 1:** İpek’s first session.

Session Name	Session Summary
**1st Session: History Taking and Therapy Planning**	According to the EMDR developmental protocol, the family-development-trauma history was obtained with the participation of the mother. This stage lasted for one session (60 minutes).
	For the desensitization phase, which will continue online, İpek’s 23-year-old sister was designated as the family member who will support her.
	The traumatic memory was identified as the moment of the car accident for the therapy plan. It was noted that triggers would also be addressed if necessary.

When collecting Ipek’s history, I focused on the history of the trauma, reasons for seeking counseling, when and how the traumatic event occurred, and the coping methods that have been used so far. Ipek’s mother, Saniye (codename), was visibly tense as she recounted the trauma. She spoke quickly while describing the car accident and seemed confused about the order of events. It was clear that she was still affected by the incident. Therefore, it was decided that in subsequent sessions, when a family member needed to accompany her, her 23-year-old sister would attend the sessions. Ipek’s developmental history revealed that she had never slept alone since infancy. She was the youngest child and was born as a desired baby after a 15-year gap between siblings. Her mother expressed self-blame for Ipek’s sleep issues, stating:

*“We tried to make her sleep alone, the child could have done it, but I guess we could not let her go. For example, when she was sleeping, I checked on her, wondered if she was tucked in, or if something has happened, and slept with her.”*

At the end of the meeting, I asked to meet Ipek. During our meeting, I noticed that when I asked Ipek open-ended questions, she struggled to answer and seemed to be deep in thought. Her facial expression also seemed dull. I recorded the following notes about the online meeting

*“It was my first online session, and I was excited, similar to how I feel during face-to-face counseling sessions. I looked forward to meeting the individual. It was nice to know that I was recording, as I was not occupied with problems such as the battery or the camera angle. Without these concerns, I could focus further on my preliminary interview questions after the first few minutes. However, poor internet connection could be a serious problem. I think I will be better able to determine this in future sessions.” (PCD, December 2nd, 2020)*

#### 3.1.2 Preparation phase

In the previous session, İpek was informed that she could attend the sessions with her sister if she wished. During this session, she mentioned that she decided to attend alone because her sister had to be at work. A summary of İpek’s second session is provided in **Table 2**. Before the exercise, I taught İpek the butterfly hug to help her discover the BLS rhythms. The butterfly hug is used for the client to provide bilateral stimulation to themselves. Therapists guide the rhythm. When the client performs the BLS rhythms at a slow pace and focuses on positive feelings and sensations, the exercise itself can be used as a relaxation technique during the preparation phase (30, 31).

**Table 2:** İpek’s second session.

Session Name	Session Summary
**2nd Session: Preparation Phase**	İpek attended the preparation phase alone.
	The butterfly hug was taught.
	The Bond of Love exercise was performed.
	The type of BLS was tactile.

**Figure 1:**
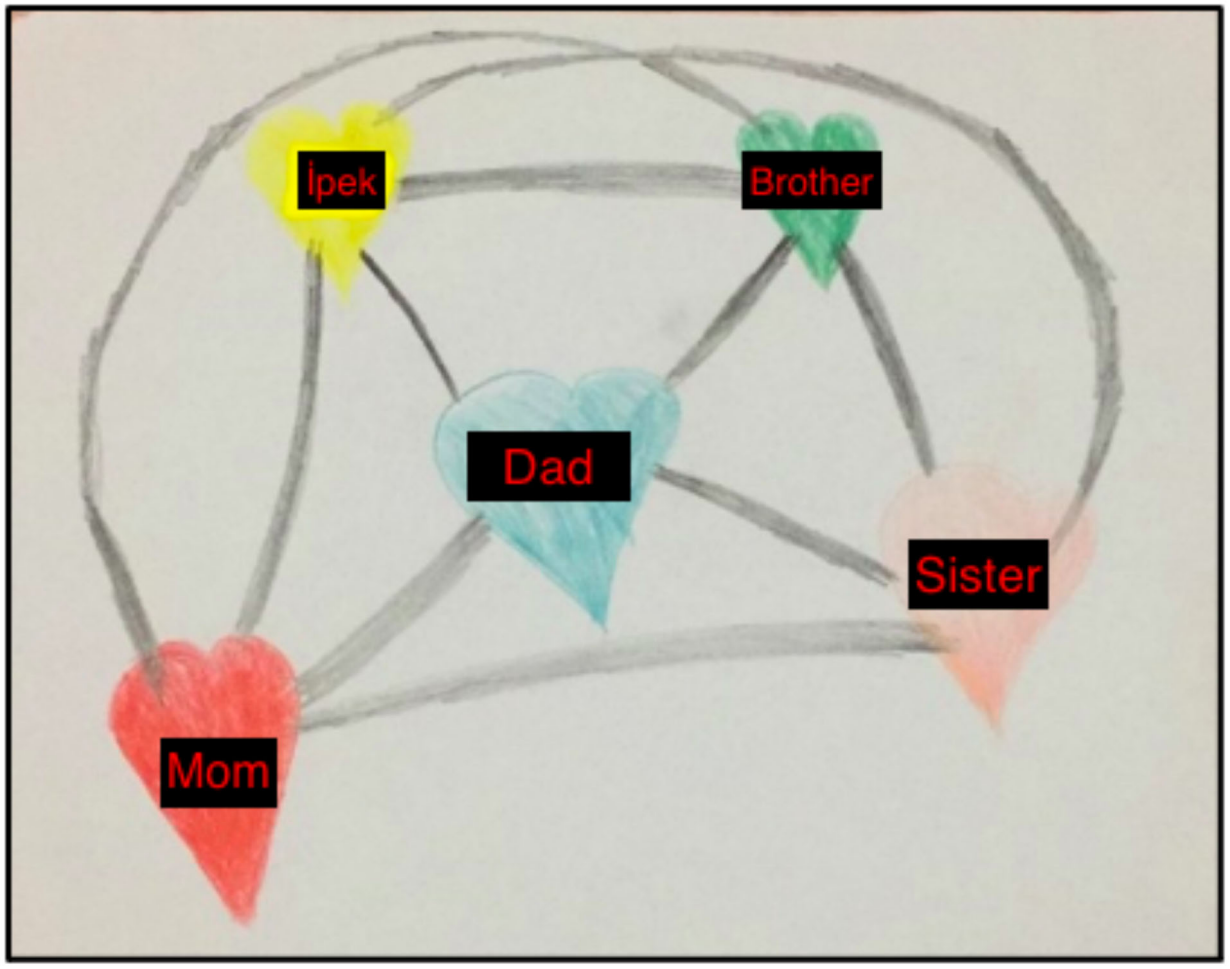
Ipek's Bond of Love Drawing – Second Session, December 4 2020.

In the preparation session with Ipek, I wanted to focus on stabilization through the “bond of love” exercise (32). I asked the parents to provide materials such as crayons and blank sheets or a picture book. To begin the exercise, I asked Ipek to draw a heart in any size and color, and in any location on the page. Then, I asked her to draw another heart for people or things she loves. These could be places where she feels safe, things that are good for her, or things that provide comfort. Lastly, I asked her to draw the bonds of love between these hearts. I emphasized that she could include not only safe people but also safe places in the exercise. However, Ipek chose to only draw family members (**Figure 1**).

When I asked İpek how she felt when looking at the picture she drew, she answered, “Good and happy.” When I asked where in her body, she felt these emotions while focusing on them, she replied, “In my heart and arms.” Following this, I asked her to focus on all these positive feelings and perform BLS at a slow rhythm. After 4-5 sets of BLS, she paused, took a deep breath, and said she felt better.

*“The most challenging aspect of the preparation phase in the online process was not being able to use my materials in the office. If İpek had not been able to do the bond of love exercise, I needed to have a plan B. I think it’s very important to consider the materials, communicate with the family beforehand, and control the process to ensure there are no deficiencies.”*

These PCD notes highlight the importance of involving the family in the preparation phase in the online process.

#### 3.1.3 Assessment, desensitization, installation, body scan and closure phases

Including a family member in sessions during the desensitization phase is important when working with children one. This family member acts as a co-therapist during bilateral stimulation and the exchange of drawing papers (33). In Ipek’s case, her older sister, mother, and father were present during the accident. Ipek’s older sister, Oya (code name), had previously received adult EMDR therapy for depression after the accident. When the details of the study were announced, Oya applied on behalf of her younger sister, believing that EMDR would be helpful for her sister’s healing process. When discussing family members to accompany Ipek during the desensitization phase, Oya volunteered. Even though they were together during the accident, the researchers determined that it would be appropriate for them to do the session together as Oya had completed her own EMDR therapy sessions previously.

During the assessment stage, Ipek drew the most distressing image related to the accident on a piece of paper smaller than A4 size and made other evaluations based on her drawing. I instructed her to write down any negative thoughts and feelings about the event as she began to draw, in order to determine her current disturbing emotions and negative cognitions (NCs) related to the traumatic memory. Ipek was unable to identify any NCs and instead stated “I am safe, it was not my fault” as positive cognitions (PCs). She reported feeling afraid and sad, and experiencing itching sensations in her arms. Her Subjective Units of Disturbance (SUD) score was 5. Once the initial drawing was ready, I instructed Oya, “While Ipek focuses on and looks at the drawing and concentrates on the distressing feelings, body sensations, and other aspects, you will perform BLS with a fast rhythm, one on the right shoulder and one on the left shoulder.” I also told Ipek, “If you feel too distressed and feel you can’t continue, you can make a stop sign with your hand,” and then we started the BLS.

**Figure 2:**
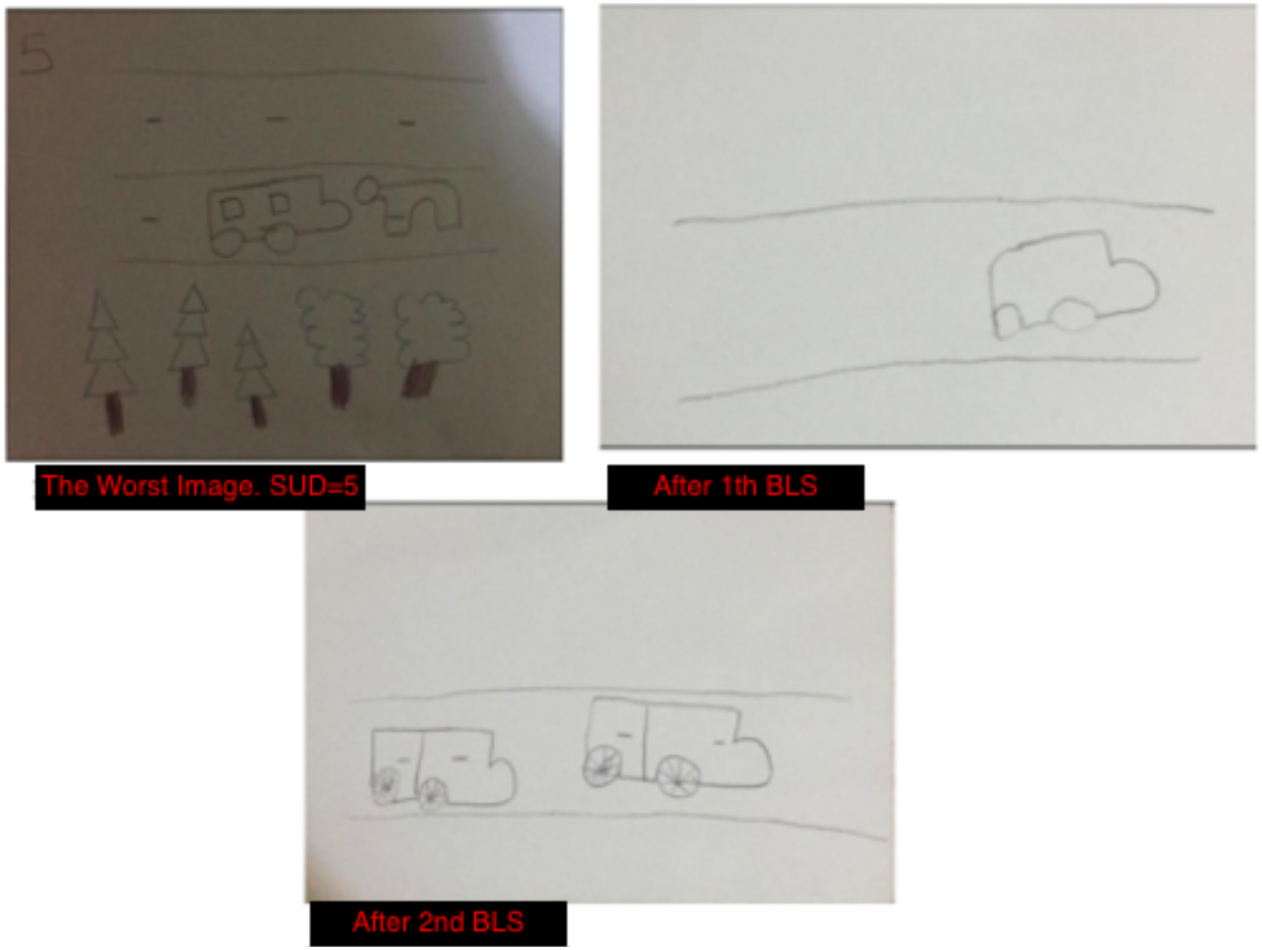
Ipek's Drawings in the Desensitization Stage.

BLS touches continued for about 30 seconds. This means approximately 16 bilateral touches were completed. When the first set was completed, I asked Ipek what she felt and instructed her to draw whatever came to mind. After the drawing was finished, I instructed Oya to begin BLS again. After 30 seconds of BLS touches, the second BLS set was completed, and Ipek drew whatever came to mind again. Then Ipek stopped the process and reported a SUD score of zero. The installation phase was conducted based on her positive beliefs of “I am safe” and “it was not my fault.” Focusing on the memory and positive beliefs, her sister continued with BLS touches for another 30 seconds. After 2 sets, her beliefs were stronger, and her VoC score was 7. During the body scan phase, Ipek expressed feelings of happiness in her arms and toes. The change in her itching sensations in her arms before the traumatic memory indicated the success of the process (**Figure 2**).

Furthermore, during the sessions, Ipek was able to name her emotions without much hesitation when asked how she was feeling, indicating that changes were taking place during the sessions. I was able to conclude that the third counseling session with Ipek was a completed EMDR session, as we had finished the installation and body scan stages. The diary entry after this session read:

*“Ipek slept alone for the first time. That is incredible” (Sister’s diary, December 11th, 2020)*

showing a reduction in post-traumatic symptoms.

The PCD notes addressed the following regarding the use of EMDR with children online:

*“In the online use of EMDR with children, it is quite meaningful to have a reliable guardian from the family participate in the sessions, as it provides both a relational resource and the structured progression needed, requiring the parent to act as a co-therapist. It is essential to ensure that the internet does not freeze or disconnect during the desensitization session. Testing beforehand will be valuable. I asked them to arrange the screen so that I could see both the sister and Ipek. It was important for me as the therapist to be able to control both the speed and the sets of BLS, with the sister providing the stimulation by touching Ipek’s shoulders and performing the BLS.”*

#### 3.1.4 Closure sessions

In the fourth session with Ipek, we reviewed the progress made during previous sessions and focused on future work. **Table 3** provides a summary of the termination sessions.

**Table 3:** İpek’s closure sessions.

Session Name	Session Summary
**4th Session**	The SUD levels of the triggers were reported as zero.
	Her future dreams and hope.
**5th Session**	Termination session She attended with her sister.

White cars and traveling by car were triggers for Ipek. Since the SUD scores Ipek assigned to both triggers were zero, a session focused on Ipek’s dreams for the future and a sense of hope was conducted before ending the meetings. It was considered important to develop Ipek’s sources of positive emotions. I believed that it would be beneficial to focus on her future goals and aspirations, and we continued to work on this issue. Ipek shared her dreams of becoming a better violin player, having a pet bird, and improving her jumping rope skills through drawings. In the fifth session, we ended the counseling sessions. Ipek attended the session alone, her older sister joined later, and we said our goodbyes. Ipek reported feeling good and not thinking about the accident since our last session, and her facial expressions seemed happier. They had been unable to travel due to the COVID-19 pandemic, but her dreams of traveling no longer troubled her.

The PCD notes identified the following as the main challenges in the online EMDR sessions with Ipek:

*“It was difficult not being able to see the client’s drawing when using the drawing method. Since the computer screen can only look at a fixed place, it was also hard to observe the client’s body sensations. If the client had joined the session through a tablet, it might have been easier to show the drawing or she wouldn’t be limited by the camera’s field of view when she wanted to move.”*

#### 3.1.5 Follow-up session (one month later)

After completing five EMDR-based counseling sessions with Ipek, I wanted to evaluate whether she had changed. In the follow-up session, I first asked Ipek’s mother to share her observations of Ipek and the process. The session transcript was as follows (Follow-up Interview, January 31st, 2021):

*Counselor: Can you share what you have observed in Ipek, both about our process and Ipek’s changes?Mother: I have observed that nothing has regressed, in fact, things have improved. For example, Ipek can now stay alone at home. I used to never leave her alone, but I was able to leave her alone for an hour and she was fine. She used to always need to stay with our downstairs neighbor, but now she can stay by herself. Also, she can sleep alone in her own bed now, which is great. We were also able to travel in our own car for 1.5 hours without any problems. I am very happy with the progress we have seen and want to thank you for your help.*

After discussing with the mother, I met with Ipek. She looked happier than ever and in her latest drawing practice, she included her pet bird and violin as hearts, unlike her previous drawings.

Ipek’s CRIES pre-test and post-test and follow-up item scores are presented in **Table 4**. By comparing Ipek’s pre-test and post-test and follow-up scores on the Child Revised Impact of Events Scale (CRIES), I observed that the scores for all 8 items decreased in the follow-up test. Before the psychological counseling sessions, Ipek was not able to travel in others’ vehicles, preferred not to talk about the incident, and had difficulty calming down due to images of the accident in her mind. After the psychological counseling sessions, she no longer had unwanted memories of the traumatic event and did not have to actively try to push them out of her mind. The fact that Ipek responded “not at all” to these items in the follow-up test demonstrates the permanence of the changes.

**Table 4:** CRIES pretest-posttest and follow-up item scores for Ipek.

Item	Pre-test	Post-test	Follo-up test (one month)
I1- Do you think about it even when you don’t mean to?	Rarely	Rarely	Not at all
I2- Do you try to remove it from your memory?	Sometimes	Not at all	Not at all
I3- Do you have waves of strong feelings about it?	Rarely	Not at all	Not at all
I4- Do you stay away from reminders of it (e.g. places or situations)?	Rarely	Not at all	Not at all
I5- Do you try not talk about it.	Sometimes	Not at all	Not at all
I6- Do pictures about it pop into your mind?	Often	Rarely	Not at all
I7- Do other things keep making you think about it?	Rarely	Not at all	Not at all
I8- Do you try not to think about it?	Often	Rarely	Rarely
Total score	22	3	1

* Not at all =0, Rarely=1, Sometimes=3, Often=5

### 3.2 Eylül’s sessions

#### 3.2.1 History taking and therapeutic planning

Only Eylül’s mother, Gülşah (codename), attended the history taking session. She appeared attentive and focused. I began taking Eylül’s history of traumatic life events, and after Gülşah recounted her experiences, I asked about Eylül’s development history. While discussing Eylül’s infancy, Gülşah seemed pleasant and smiled. However, when I asked about her relationship with her husband and Eylül, I noticed that Gülşah’s speech became more subdued. Her description of herself as a “person of duty” when talking about their relationship indicated that she may have had difficulties expressing her feelings towards her husband and child. I met Eylül during the last ten minutes of the session. She learned that I had spoken with her mother and that I would be speaking with her as well. It was identified as a goal for Eylül to overcome the feelings of withdrawal she experienced after her grandmother’s death. However, since Eylül did not have a clear memory of the loss, the therapy plan included the storytelling of the loss and the desensitization phase using EMDR’s healing story technique. The participation of the mother was also requested for all sessions.

#### 3.2.2 Preparation stage

The preparatory phase for Eylül was planned to be conducted over two sessions. A summary of these sessions is shown in **Table 5**. A 15-minute video call with the mother was conducted the day before the preparation phase sessions. During this call, the exercises to be performed in the sessions were introduced, information about BLS was provided, and how to perform shoulder taps for Eylül when needed was explained. It was considered that 10-15-minute pre-session meetings with family members, conducted after the sessions with İpek, would contribute to the process and facilitate online meetings.

**Table 5:** Eylül’s preparation phases.

Session Name	Session Summary
**1th Session**	Butterfly Hug for BLS The Bond of Love Exercise She attended with her mother
**2nd Session**	Memory Sack Exercise She attended with her mother
**3rd Session**	Auditory BLS Circle of Loving Exercise

**Figure 3:**
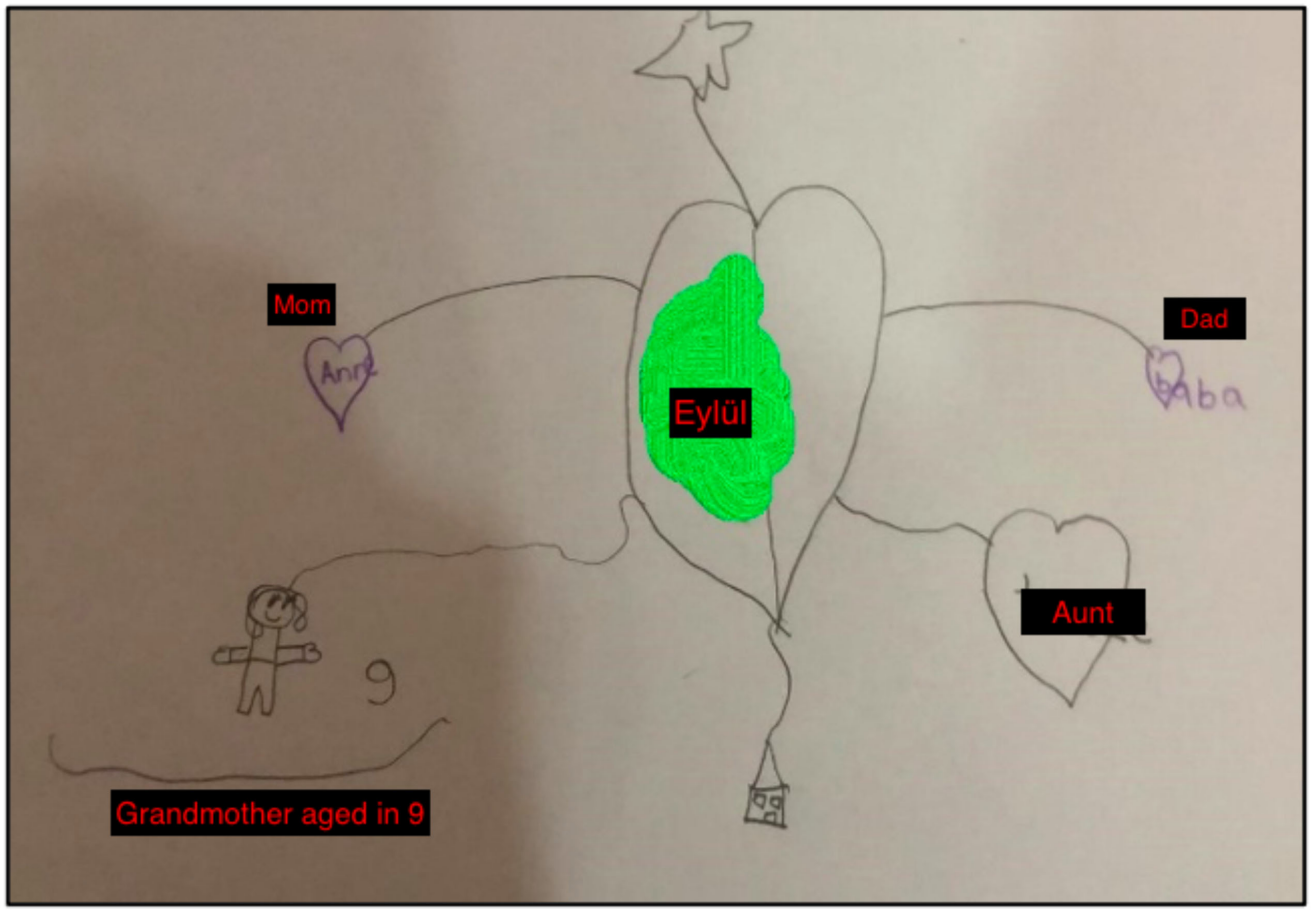
The Bond of Love Drawing by Eylül.

In the first session, I asked Eylül to get out crayons and paper for the bond of love exercise. During the exercise, she was able to communicate and express herself better. The butterfly hug was taught to Eylül so she could perform tactile BLS. After drawing her picture, Eylül focused on the positive feelings and bodily sensations she experienced and performed the butterfly hug with 5-6 bilateral taps. The **Figure 3** was drawn by Eylül. The entries in her mother’s diary summarized the first session with Eylül:

*“She was a little nervous before the session, but after getting to know you and starting the activity, she was happy. After the session, she told me, ‘I felt my grandmother. When I was hugging the butterfly, my grandmother was on my mind.’ I think the session was good for Eylül. During the week, she asked me 4-5 times: ‘Can we do a butterfly hug?’ She said, ‘Mom, this relieves me’ during the butterfly hug” (Mother’s diary, January 26th, 2021).*

**Figure 4:**
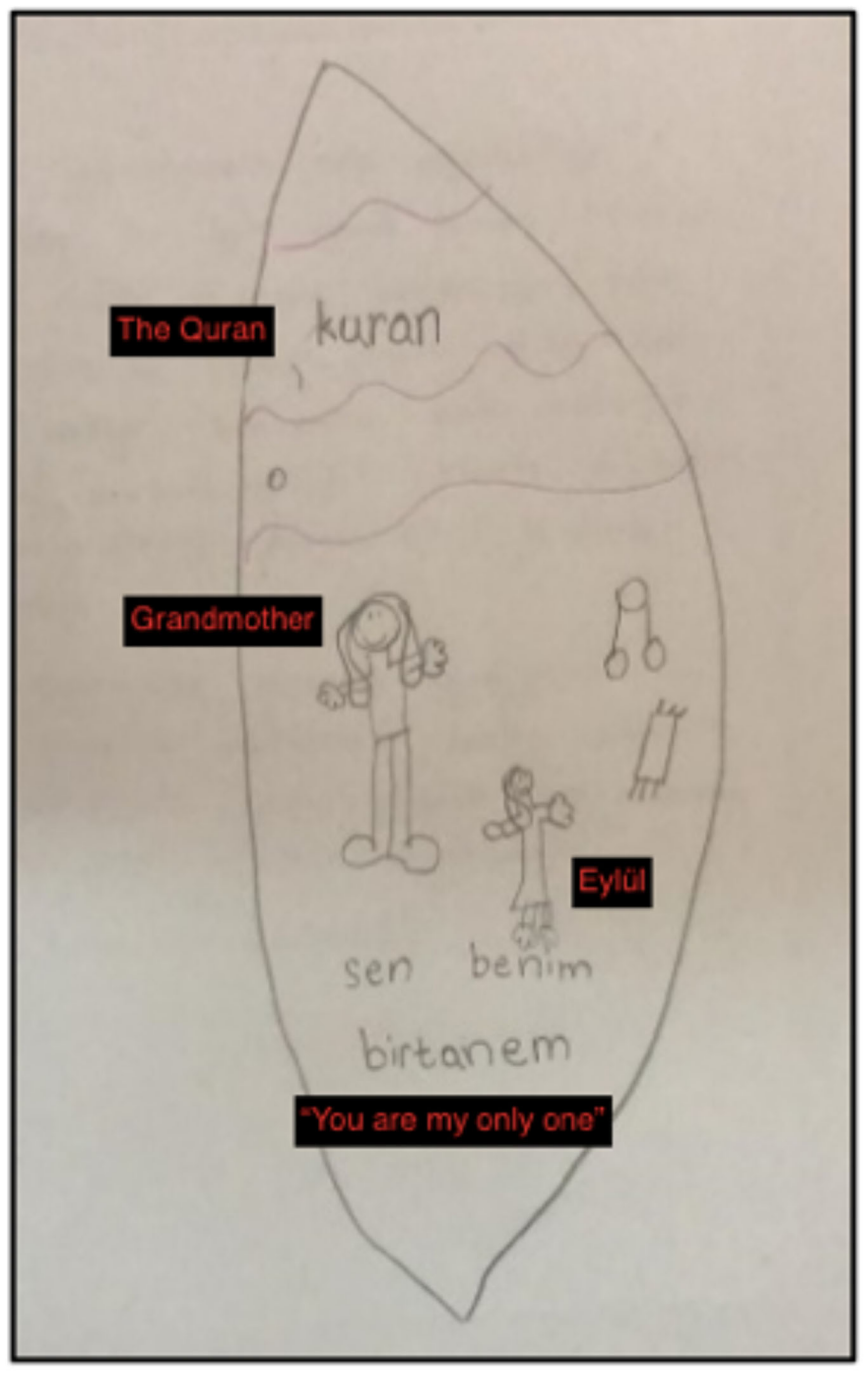
Memory Sack Drawing by Eylül.

In the second session, I began by reviewing the previous week with Eylül. Knowing she had used the butterfly hug, I wanted to ensure she was doing it correctly. When working with children, it is essential to use resource development/installation activities during the preparation phase (34). Here, we used positive memories between Eylül and her grandmother as a relational resource. Eylül was asked to think of positive memories of her grandmother and imagine or draw them as if she was collecting them in a sack. Then, I helped Eylül develop resources by focusing on the positive thoughts, emotions, and body sensations associated with each memory and by doing 2-4 sets of self-administered BLS (butterfly hug). I started the memory sack exercise by referring to the bond of love exercise, since she thought about it during the butterfly hug (**Figure 4**).

*Counselor: Now I want you to think about it, let’s find the memories that make you feel happy and pleasant when you remember your grandmother and let’s draw a picture or figure for each of these in the sack. If you don’t want to draw a picture, you can write a word or a sentence that describes that memory, however you want, so that you can easily remember it. When you decide, you can share it with me.*

*Eylül: I will tell you when I am done (Starts drawing something) (Second Session, January 27th, 2021).*

During the online EMDR study, I shared my notes on resource development and installation on the PCD:

*“Eylül and her mother joined the Zoom sessions using a computer. I believe the greatest challenge of the online sessions was the difficulty in following the drawings. To address this, I asked Eylül to describe what she was drawing while she worked on her sketches.”*

*“Today’s session was a lot of fun and it was very nice to put the good memories about my grandmother in the sack. I have good memories about my grandmother and that makes me happy. It was very nice when I went on a picnic with my grandmother, she taught me the Qur’an and she hugged me like my own mother, and when I think about this, I am happy” (Mother’s Diary, Eylül’s words, January 27th, 2021).*

After the client’s feedback, I once again realized the value of the memory sack exercise during the preparation stage.

In the third session, the Circle of Loving exercise was conducted (**Table 6**). This exercise is used in attachment-focused trauma and for clients dealing with feelings of grief to help them feel peace, safety, and reconnect with a sense of bonding. While Eylül was performing this exercise, she didn’t want to draw; she preferred to imagine instead. As an alternative to tactile BLS that her mother could provide or the butterfly hug that she could do herself, I had an auditory BLS as a backup plan for the sessions. For this, I asked her mother to have a working pair of headphones available during the sessions. I also had instrumental music on my computer, known as 8D music, where the sound rises in one ear and then the other, providing bilateral stimulation. Since we were going to do resource work, I started the process with a slow-paced piece of music. Eylül could hear both me and the music, so we didn’t encounter any difficulties during the online exercise.

**Table 6:** İpek’s third session.

Session Name	Session Summary
**3th Session: Assessment, Desensitization, Installation, Body Scan and Closure Phases**	Target Image: Moment of the Accident NC: Unable to Identify SUD: 5 PC: It’s Not My Fault VoC: 4 Emotion: Fear Body Sensation: Tension
	Desensitization was carried out by İpek’s sister with compassionate touches on İpek’s shoulders, thus using tactile BLS.
	When SUD reached 0, the PC “It’s not my fault, I am safe” was installed. During the body scan, no negative sensations were reported, and the emotional expression was described as relaxation.

#### 3.2.3 Desensitization and installation stages

When working with children, it is important to prioritize a focus on the developmental protocol, the developmental needs of the child, and develop a treatment plan accordingly (35). Eylül was more suitable for EMDR due to her improved storytelling technique and her age. The healing story is based on the narration of the traumatic event experienced by the child with an animal/figure that the child can identify with. Past, present and future are discussed in the story. It is important to start the story with a positive note based on strong and safe sources from the past. Then, the traumatic event could be described. The narration of the traumatic event ensures the assignment of new meanings to neural networks. Thus, it could also include the installation stage. In the conclusion stage, the positive perceptions about the future are emphasized. The story is read to the child with bilateral stimulation. In order to include a healing story with children, the story should be read to the parent with bilateral stimulation and the parental SUD is expected to be below 5 (36). Gülşah’s story SUD was 3.

Eylül could not remember certain events related to the loss of her grandmother. However, she had difficulties in understanding the loss and exhibited introverted behaviors as a result of having lived in the same house during her grandmother’s illness but not being told of her passing. In the desensitization and installation stages of working with Eylül, we used EMDR with a healing story approach. This approach is based on the narration of the traumatic event experienced by the child with an animal/figure that the child can identify with. The story is designed to start positively, include traumatic events, negative emotions, and sensations, and then end positively with coping strategies and expressions of hope (10, 33).

A healing story titled “The princess who learnt to grieve” was written by the researcher and read to Eylül while her mother performed bilateral stimulation by touching her shoulders. Eylül’s level of distress as measured by the Subjective Unit of Disturbance (SUD) was 7 before the story was read, but decreased to 1 after the story was read. She reported feeling relaxed and longing for her grandmother instead of unhappiness. She also noted that she will always miss her grandmother, even though she is no longer alive. After I finished reading the story, I wanted to determine whether Eylül identified with it. The related transcript is presented below:

*Counselor: You can stop now. How was the story Eylül?*

*Eylül: I realized that, like me, it happened to my grandmother as well, now I realized it.*

*Counselor: So, is there anything in this story that you would like to change or add?*

*Eylül: I mean, since my grandmother died and also her grandmother died in the story, and now that I think about it, I do not want to add anything because the story is beautiful.*

*Counselor: So, what I’m asking you to do is to draw a picture of whatever was the best for you in the story. If more than one thing was good, you can draw what was the best, or you can divide your page, you can draw more than one thing. ^1^*

*Eylül: Huh, I fold it once so I could draw two. (She points to the paper).*

*Counselor: Great, good idea.*

*Mother: She wrote a few things, she wrote a butterfly hug, she wrote love. Now she’s drawing something. On the top, it reads butterfly hug. It reads love at the bottom.*

*Counselor: Who are those below, there are two drawings?*

*Eylül: These are my grandmother and me, I dyed our hair the same color to look a little bit similar.*

*Counselor: Which one is you and which one is your grandmother?*

**Figure 5:**
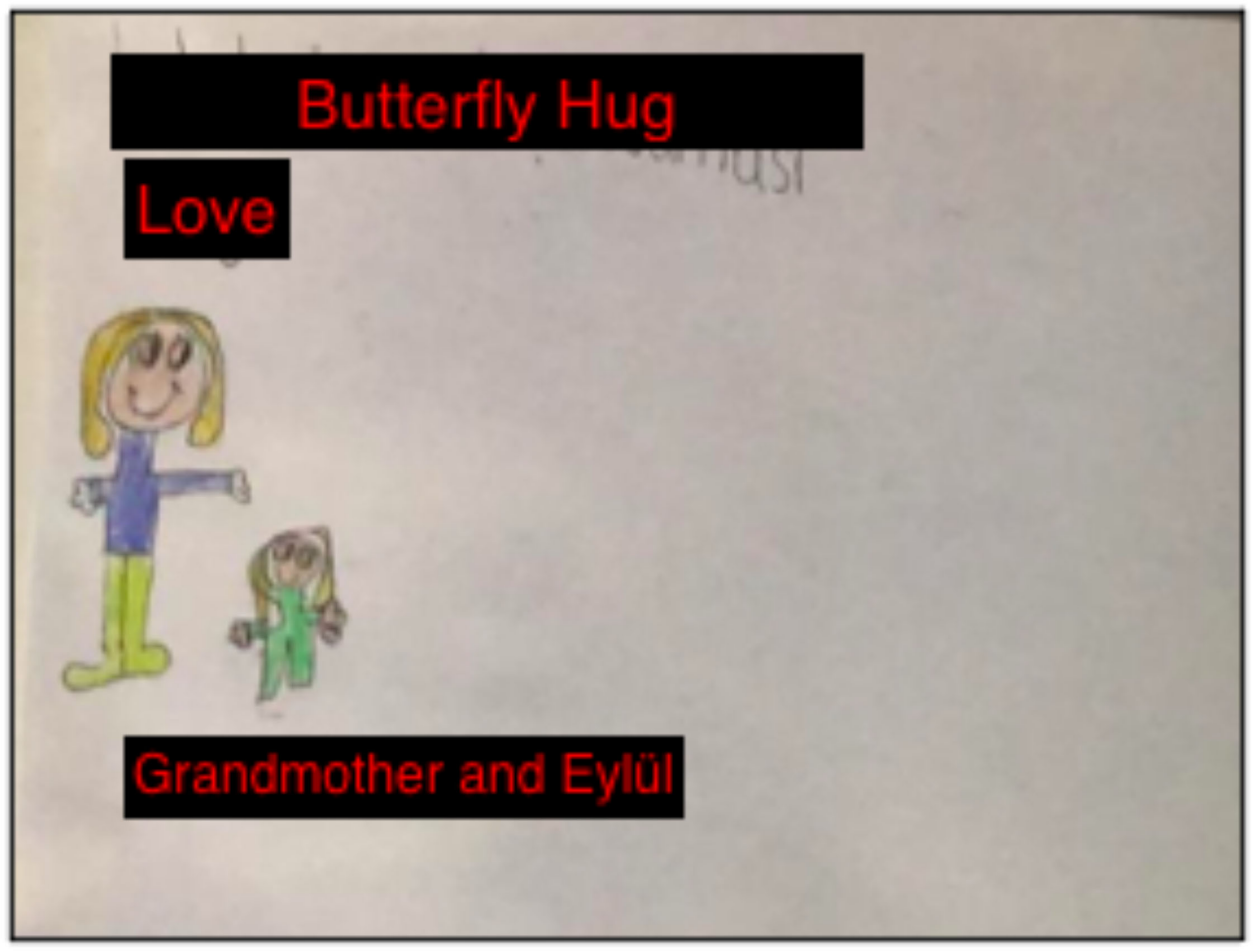
Post-Healing Story Drawing.

*Eylül: The small one. The little one is me; the big one is my grandmother. (**Figure 5**, Fourth Session, February 20, 2021).*

The healing story is a unique approach for addressing trauma in children, as it helps them integrate their implicit and verbal memories. According to Lovett (37), the healing story can aid in resolving trauma, strengthening the bond between the child and caregiver, and developing emotion regulation skills. This technique was successful in helping Eylül distance herself from her trauma, confront her loss, and improve her coping skills.

After completing the desensitization and installation stages, Gülşah recorded positive developments in Eylül’s sleep in her diary as follows:

“After the session, Eylül did not want to talk about the story anymore. She was a little nervous but wanted to sleep together before going to her room. I went to her room and kissed her, she slept alone and never woke up until morning. She did not wake up for 2 days at night.” (Mother’s Diary, February 22nd, 2021).

*“I believe that involving Eylül’s mother in the session made the online process smoother, particularly during the desensitization with the story. The mother had no difficulty with the process as she understood how to do BLS. However, it was challenging to read the story on the screen and to follow the postures and movements of Eylül and her mother simultaneously. In such cases, it may be important to devote more time to the body screening phase.” (PCD, February 22nd, 2021).*

#### 3.2.4 Closure sessions

**Figure 6:**
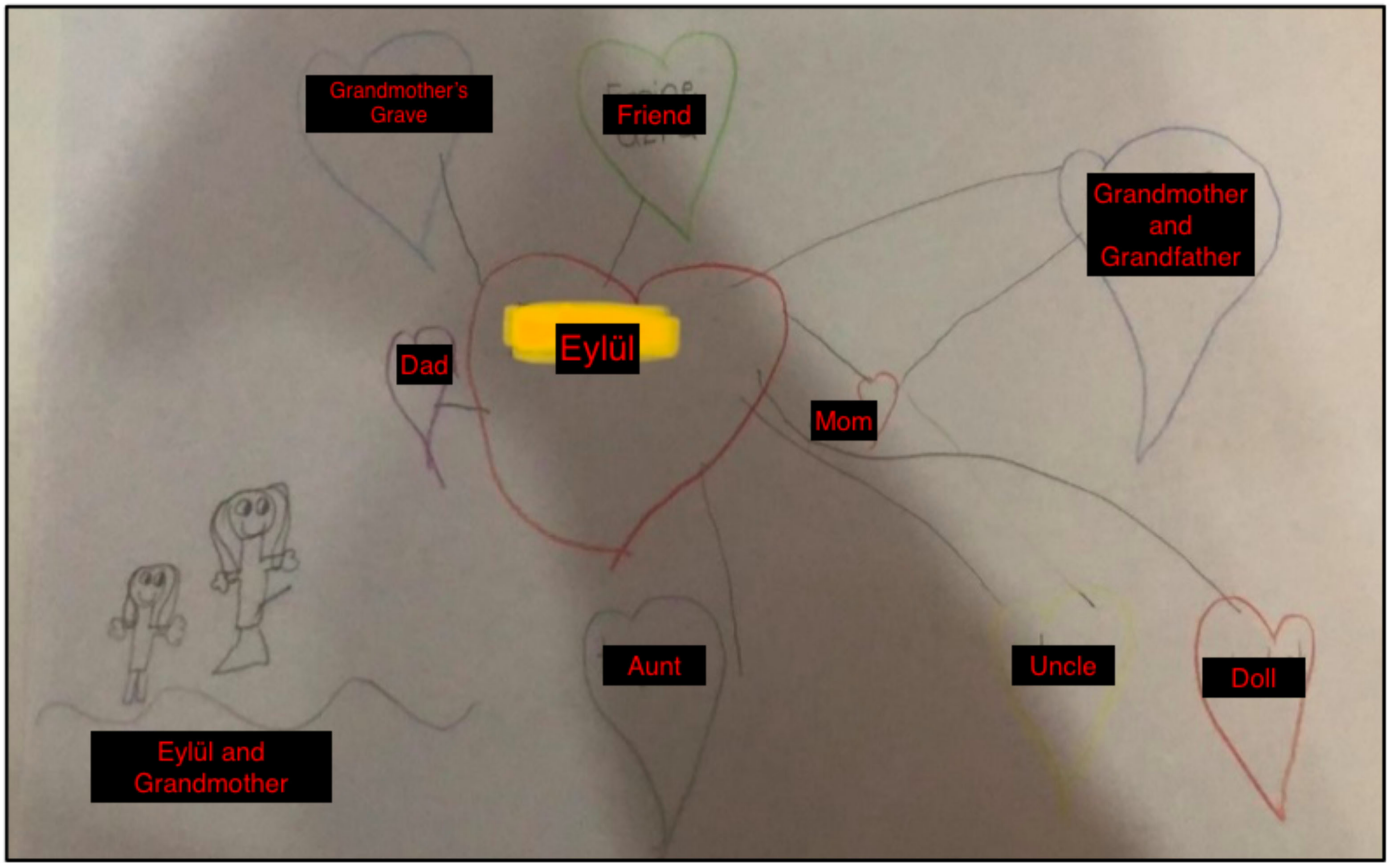
Eylül's Bond of Love Drawing in the Fifth Session.

Fifth session: The closure stage of EMDR therapy with Eylül lasted for four sessions. In this section, two sessions are described to illustrate the assessment of the EMDR process. In the fifth session, Gülşah noted that one of the reasons they sought psychological assistance for Eylül was her reluctance to speak about her grandmother, her reluctance to share her thoughts and her introversion. In this session, Eylül reported that she now felt more comfortable speaking. The therapist also assessed Eylül’s progress by repeating the bond of love exercise. The **Figure 6** was drawn by Eylül.

The changes in Eylül’s bond of love drawing were significant. The most striking change was the larger size of the drawing of her grandmother. In the first exercise, she had drawn her grandmother as a nine-year-old child, but now she was able to depict her in adult form. This change was seen as a sign of the therapy’s success. Another notable change was the inclusion of friends’ names in addition to family members, indicating that her social resources had begun to improve. Additionally, the inclusion of a doll her grandmother had made for her among the hearts, or positive memories, indicated that her resources had become stronger. The only aspect that was confusing was the inclusion of her grandmother’s grave within a heart.

Although Eylül’s statement that “When we visit her grave, I feel peaceful there” did not convey a negative message, the therapist was unsure of how to interpret the drawing. The therapist also discussed this with her supervisor:

*“If the grave has a positive meaning in the child’s life, it may be appropriate to leave it in the drawing, but if it has negative connotations, it may be beneficial to suggest an alternative. It is important to discuss this with the child and consider the impact of the image on their life. The fact that the grandmother grew in size in subsequent drawings is an indication of progress in therapy” (Supervision Notes).*

Ninth Session. Eylül’s expressions of change during this session were noteworthy. Her statements, such as “they converge in my head”, “they match with me”, “they seem to enter my heart” confirm that there was activation between her right and left brain.

**Figure 7:**
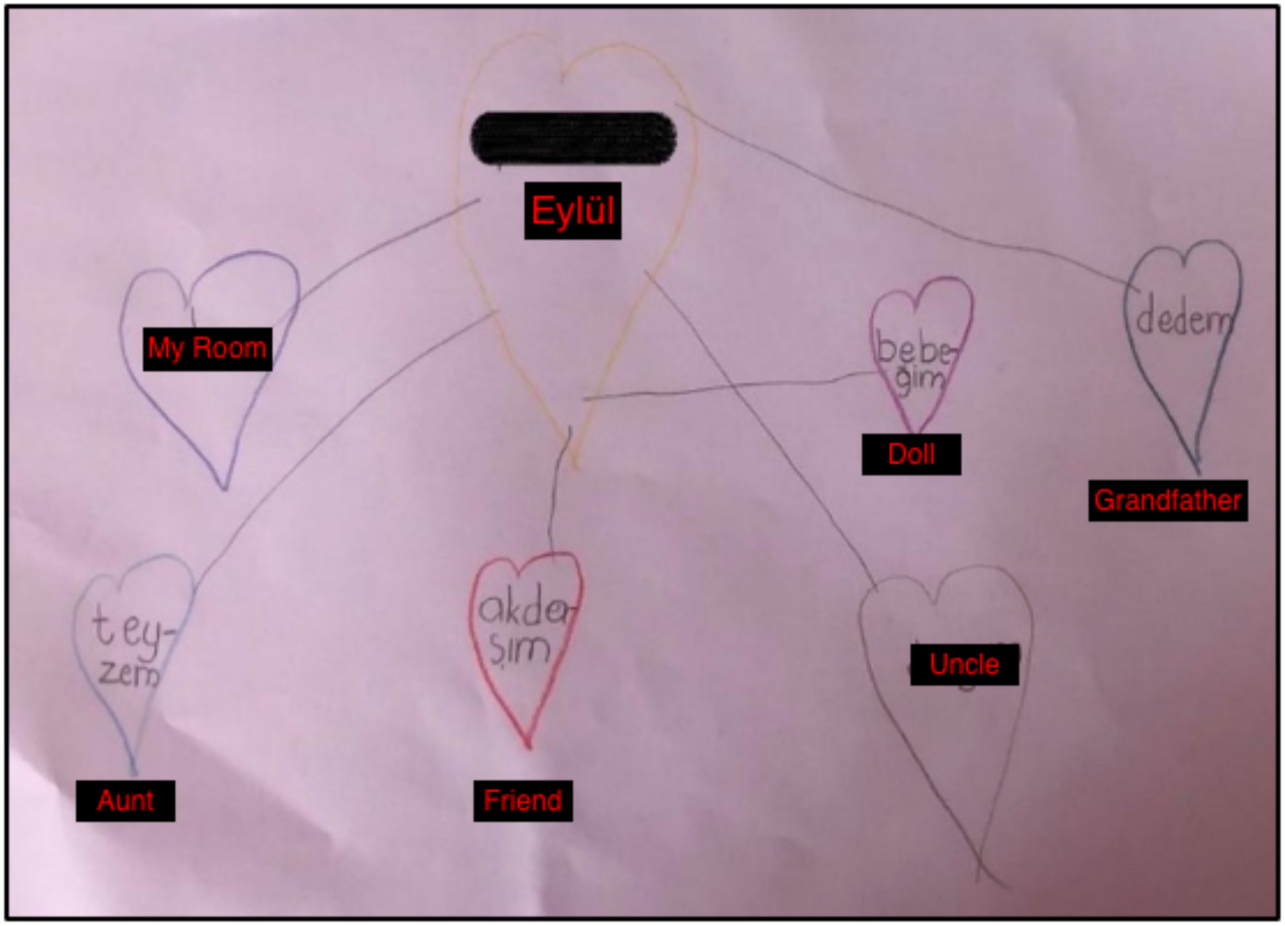
The Bond of Love Drawing by Eylül (Final Session).

*Eylül: I did not write my mother, father and grandmother on purpose because they are inside me. My grandma may be far away, but she is inside of me (* **Figure 7** *).*

Counselor: Come on then, look at those hearts once more and do the butterfly hug.

*Eylül:* (She does, she takes deep breaths). *I want to say something. It is like the pieces match up in my brain. Parts matched. When I said everyone matches with me, I said, it was like they entered my heart (Ninth Session, March 26, 2021).*

#### 3.2.5 Follow-up session (one month later)

During the follow-up session, Eylül was visiting her grandmother’s hometown with her parents, grandfather, and aunt. Being at the location of her grandmother’s grave provided an opportunity to assess any changes or triggers Eylül may have experienced. During the session, Gülşah reported on the improvements she and Eylül had made and the coping skills Eylül had retained:

*Mother: “We are doing better now. For example, we never used to play together and build things, but now we play games more, or when I am with her, we can build a house or play in her tent. After the sessions, I can honestly say that the process was beneficial for me as well. Eylül once said, ‘Mom, I was so bored, I was so nervous, I did a butterfly hug,’ but I did not see her do it, she did it on her own. She told me later.” (Follow-up session, May 1, 2021).*

**Figure 8:**
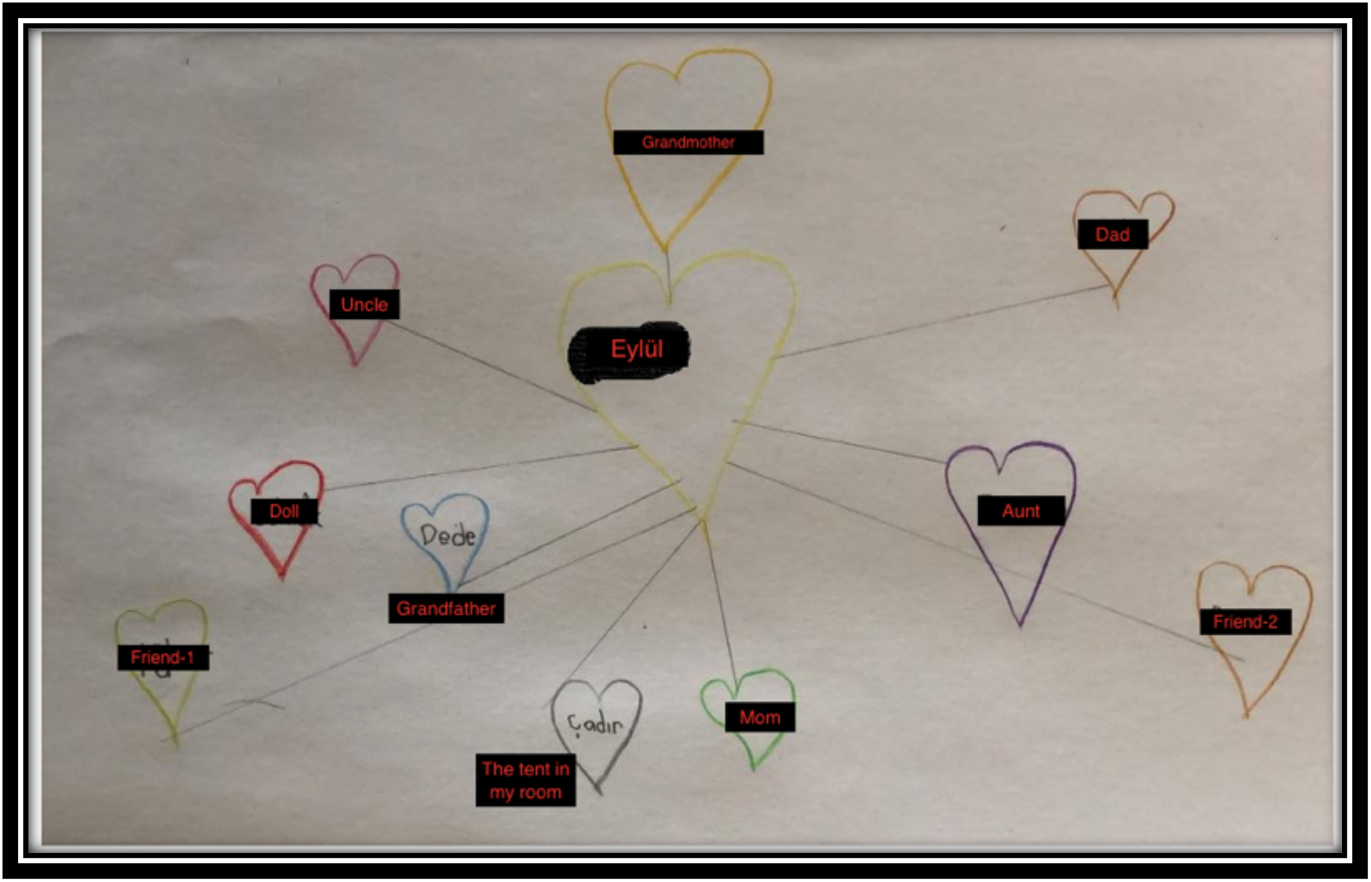
The Bond of Love Drawing (Follow-up Session).

Before ending the session, the therapist wanted to repeat the bond of love exercise to observe the changes after the closure session. Eylül remembered the bond of love exercise, so the therapist briefly repeated the instructions and gave her some time to draw. The **Figure 8** was drawn by Eylül.

In this session, Eylül was able to draw her grandmother not as a figure in a grave, but as a loved one. She also depicted the tent, which symbolized her family, friends as social resources, and calming skills as internal resources. This suggests that her coping skills with her grandmother’s death have improved and she is enjoying social relationships. Despite her high cognitive abilities, it was important for her to be social, have fun, and be a child. The inclusion of her friends in the hearts was a positive sign. The therapist is proud of Eylül for her courage and dedication since the first session.

The comparison of Eylül’s pre-test, post-test, and follow-up Child Revised Impact of Events scores revealed that her scores in all 8 items continuously decreased in the follow-up test. Eylül’s CRIES-8 pre-test total score was 21, her post-test score was 6, and her follow-up test score was 4. The analysis of the item scores showed that she responded often (5 points) in item 3 (Do you have waves of strong feelings about it)?, item 5 (Do you try not talk about it.), and item 7 (Do other things keep making you think about it)?, however, her responses to these items were not at all in the follow-up test. As Gülşah mentioned in her diary, calming skills and butterfly hug may have helped Eylül to deal with intense emotions. Additionally, due to the desensitization stage, Eylül’s pre-test scores in item 6 (Do pictures about it pop into your mind)? and item 4 (Do you stay away from reminders of it) changed from Sometimes=3 to not at all=0 in the follow-up test.”

## 4 Discussion and conclusion

The present study aimed to investigate the use of online EMDR in children with single-incident trauma and evaluate its impact. The study detailed the online EMDR sessions conducted with two children, aged 6 and 8.

I conducted five EMDR-based counseling sessions with Ipek, including the initial session, the session with her mother, and the follow-up session. Although Ipek initially exhibited poor facial expressions and emotional responses, she actively participated in the sessions and successfully overcame her difficulties. She can now sleep comfortably alone and even joke about the memory of the accident. Teaching bilateral stimulation techniques, resource development, and the desensitization phase were crucial during the EMDR sessions with Ipek. The observation of Ipek’s weak emotional expressions and her potential difficulty in following instructions during the initial session highlighted the critical importance of having a family member actively participate in the sessions. This family member worked as a co-therapist during the BLS touches, which proved to be crucial for the online EMDR sessions. I conducted ten EMDR-based counseling sessions with Eylül, struggled to cope with her grandmother’s death. She was behaving older than her age, had sleep difficulties, and avoided discussing death. After undergoing EMDR-based psychological counseling, her sleep problems were resolved, and she became more comfortable discussing her emotions and thoughts about her grandmother’s death. She gained insight into the grieving process and found answers to her questions about death. Through the use of calming skills, she also strengthened her relationship with her parents.

A systematic review of online EMDR applications for post-traumatic stress disorder by Lenferink et al. (16) found only one study conducted during the COVID-19 pandemic and highlighted the need for further research. Although this study does not aim to measure the effect size, it serves to describe how online EMDR sessions are conducted with children. It can be said that the most significant challenge of online EMDR sessions is deciding how to incorporate BLS into the process. Spence et al. (38) investigated the effectiveness of a 6-week therapy program that combined a web-based EMDR device and cognitive behavioral interventions. Bilateral stimulation was conducted with a web-based EMDR device controlled by the client, but the study acknowledged the uncertainty of its effectiveness compared to face-to-face EMDR due to limited therapist involvement. Waterman and Cooper (39) conducted a meta-analysis on the use of EMDR as a self-help method and found that individuals used not only visual EMDR devices for bilateral stimulation but also audio files and tactile stimuli such as butterfly hugs. In this study, the use of tactile BLS methods stands out. The butterfly hug was used as a self-BLS method for children. Additionally, family members participating in the sessions performed BLS by touching the children’s shoulders. Auditory bilateral stimulation was also used in the EMDR sessions with Eylül’s preparation phase. Herkt et al. (40) found in a study that EMDR stimulation facilitated emotional access through neurological means, and auditory stimuli led to an increase in the activation of the right amygdala, suggesting a positive effect on the treatment of traumatic memories similar to visual stimuli. This demonstrated that the preference of bilateral stimulation in the study was consistent with the literature. According to AIP, the human brain and nervous system have an innate ability to process emotions, cognitions, and bodily sensations that arise in response to potentially traumatic events. However, when the trauma exceeds this capacity, unprocessed memories are stored in the brain in a dysfunctional format, causing ongoing distress in the child’s life. EMDR therapy addresses this by having clients focus on specific traumatic memories while the therapist applies eye movements or other forms of bilateral sensory stimulation to facilitate adaptive processing (41–43). We believe it is very important for therapists to use forms of BLS other than eye movements when conducting online sessions with children to facilitate processing. Fun and resourceful methods like bilateral tapping on the chest like a gorilla or bilateral jumping on both feet can be incorporated into the preparation phase. Civilotti et al. (44) also recommend more frequently changing different types of BLS during the desensitization phase to keep the child actively engaged.

In the research series in which they presented the effectiveness of EMDR therapy, Manzoni et al. (45) pointed out that EMDR is used more frequently with children and adolescents. Nevertheless, it is noteworthy that there is no study conducted online in the research series. Online EMDR interventions can be effective when a strong therapeutic relationship is established with clients, similar to other forms of therapy. Although there are studies showing that online EMDR interventions are as effective as face-to-face therapies (46), there is a need for more research specifically proving the effect size in this area.

In this study, online EMDR-based counseling sessions with two mid-childhood clients who experienced single-incident trauma were described using the qualitative case study method. Future research could investigate the effectiveness and permanence of EMDR sessions through various empirical methods. Additionally, further studies using neuroimaging techniques are needed to gain a more comprehensive understanding of the effects of EMDR on the brain. Further research should also be conducted with children of similar age groups to the participants in this study but with different traumatic experiences, and with larger sample sizes using group EMDR protocols for individuals experiencing similar issues. In this study, the developmental EMDR protocol was applied online; therefore, future studies could explore the online application of different protocols that could be conducted with children. Finally, in addition to the tactile and auditory BLS methods used in this research, studies utilizing web-based EMDR devices and/or smartphone applications should also be conducted.

## 5 Limitations

The study’s main strength and limitation both stem from its nature as a case study. Case studies can provide a rich contextual understanding of the information. However, they are quite weak in terms of effect size. The study found that online EMDR was effective for the presented cases, and this effect might be primarily due to the inclusion of bilateral stimulation (BLS) in the online process. This study is limited in its use of tactile and auditory BLS in online sessions. Although all sessions were conducted under the therapist’s guidance, a limitation is that the BLS was not performed directly by the therapist. Additionally, the fact that the clients participated in the sessions via computer can be noted as a limitation.

## Data Availability

The raw data supporting the conclusions of this article will be made available by the authors, without undue reservation.

## Footnotes

1 (Researcher’s Note: Here, the researcher interprets Eylül’s unwillingness to change anything as her realization that she can identify with the story and cope with the grief. It would have been beneficial to ask Eylül where she was most affected by the story to further understand her perspective and encourage her to talk more about her feelings related to the story).
